# Clinical Features Indicating the Need for Mechanical Ventilation in Patients with Guillain Barre Syndrome

**DOI:** 10.7759/cureus.5520

**Published:** 2019-08-29

**Authors:** Sumera R Umer, Qamar Nisa, Monika Kumari, Saira Abbas, Sarfraz A Mahesar, Naila N Shahbaz

**Affiliations:** 1 Neurology, Dow University of Health Sciences, Karachi, PAK; 2 Neurology, Civil Hospital Karachi, Karachi, PAK

**Keywords:** guillain barre syndrome, mechanical ventilation, bulbar weakness, facial weakness

## Abstract

Background

Guillain Barre Syndrome (GBS) is an immune-mediated inflammatory polyradiculoneuropathy. Respiratory failure is one of its recognized and most dreaded complications, requiring ventilatory assistance. Early recognition of distinct clinical predictors of mechanical ventilation may help in the better management of GBS patients in our setup.

Objective

To determine the clinical predictors indicating the need for mechanical ventilation in patients with Guillain Barre Syndrome and to compare the presenting features in patients who require mechanical ventilation and who do not.

Method

It was a prospective observational study. A total of 100 consecutive patients, over the period of one year, were included in this study. All patients were clinically examined for limb weakness, neck weakness, bulbar and facial nerve involvement, and followed up till seven days of hospitalization for whether the patient required mechanical ventilation or not. Results were recorded on a specifically designed proforma. Data were entered and analyzed using SPSS version 20.0 (IBM Corp., Armonk, NY, US).

Results

Out of 100 patients, 13% required mechanical ventilation. When clinical presentations were compared in patients who required mechanical ventilation and those who did not, a shorter interval between the onset of symptoms and the attainment of maximal disability, facial weakness, bulbar dysfunction, and neck weakness turned out to be significant factors (p-value<0.000).

Conclusion

According to these significant outcomes of our study, the course of patients with GBS leading to mechanical ventilation can be predicted on the basis of clinical presentations. So we can recommend that shorter time duration between symptom onset and peak disability, along with the presence of facial, bulbar, and neck weakness, should be taken as an indication of impending respiratory failure.

## Introduction

Guillain Barre Syndrome (GBS) is an acute-onset, usually monophasic, immune-mediated disorder of the peripheral nervous system [1]. GBS occurs throughout the world, with a median incidence of 1.3 cases/100,000 population (range, 0.4-4.0) [2]. The local incidence of the disease is unknown due to a lack of studies. Males are more commonly affected than females, and there are peaks in young adults and the elderly.

GBS may present as both demyelinating and axonal variants. Overall, the demyelinating variety is more common than the axonal but in Asian populations, the frequency of axonal varieties is much higher as compared to the western population [3-4]. Large studies in Northern China [5-7] and Iran [8] showed that the axonal forms of GBS constitute 30%-47% of patients in Asian populations. Out of four studies in Pakistan, two [3,9] showed a higher percentage of axonal variants while two [10-11] showed similar results to US and European studies.

Although plasma exchange and intravenous immunoglobulin have improved the prognosis of patients with GBS, mortality and morbidity rates remain high [12]. Several factors have been identified as predictors of poor outcome. A shorter duration between the onset of weakness and the lowest MRC score attained, along with the presence of facial and/or bulbar weakness were the main predictors of mechanical ventilation. A worse prognosis has also been identified in patients with older age and in patients with severe deficits at the beginning.

Identification of these predictors is necessary because several factors have been identified in different studies worldwide that predict the need for mechanical ventilatory support at an earlier stage of the disease. Due to the paucity of local data in this regard, we conducted this study in our setting, assuming that early recognition of these factors can have clinical, therapeutic, and financial implementations in our setup.

## Materials and methods

Study design, setting, and sample collection

It was a prospective observational study conducted in the Department of Neurology, Civil Hospital Karachi, from April 30, 2015, to April 30, 2016. A total of 100 patients were included in this study. Data of patients, who fulfilled GBS diagnostic criteria, were collected prospectively by nonprobability consecutive sampling. Patients were excluded if they met any one of the exclusion criteria: Patients with already existing peripheral neuropathy with acute exacerbation of weakness, patients below the age of 12 years or more than 60 years, those who could not undergo nerve conduction studies for any reason, e.g. patients with diffuse skin lesions or bleeding diathesis, and patients with comorbid conditions known to cause, or associated with, peripheral neuropathy.

Data collection

All adult patients who fulfilled the inclusion and exclusion criteria were enrolled. Informed consent was taken. The descriptive profile of patients was noted on an approved proforma. Enrolled patients were clinically examined for neck weakness, limb weakness, bulbar and facial nerve involvement, and respiratory muscles involvement (bedside assessment of counting in single breath and fraction of inspired oxygen (FiO2) measurement). The type of neuropathy was also recorded after electrophysiological studies. All patients were treated with either intravenous (IV) immunoglobulin or plasma exchange without any treatment delay. All of them were followed up till seven days of hospitalization for whether the patient required mechanical ventilation or not. All the results were recorded on the approved proforma.

Data analysis

Data were entered and analyzed descriptively using IBM Statistical Package for the Social Sciences (IBM SPSS Statistics for Windows, Version 20.0, Armonk, New York, US). Tables were constructed using Microsoft Excel 2016 (Microsoft Corporation, Washington, United States). Mean and standard deviation was calculated for the age and duration of symptoms from onset to peak disability. Frequency and percentage were calculated for gender, clinical presentations (bulbar involvement, neck weakness, limb weakness, and facial nerve involvement), and the need for mechanical ventilation. The chi-square test was applied to compare the clinical presentations in patients who required mechanical ventilation and who did not. A p-value less than equal to 0.05 was taken as significant.

## Results

A total of 100 patients were included in this study. The age of the patients ranged from 18-60 years. The average age of the patients was 48.1+/-8.01. Forty-four (44%) were female and fifty-six (56%) were male. The mean duration of symptoms from the time of onset to the time of peak disability was 5.6+/-3.9 days. The most common clinical features of GBS were limb weakness 95 (95%) followed by facial nerve involvement 49 (49%), neck weakness 43 (43%), and bulbar involvement 39 (39%). Out of 100 patients, 56% had axonal variety and 44% had demyelinating neuropathy on electrophysiological testing. Out of 100 patients of GBS, 13 (13%) required mechanical ventilation. Patient characteristics are shown in Table 1.

**Table 1:** Characteristics of enrolled patients (n=100)

	Mean	SD
Age in years	48.1	8.01
Duration of symptoms in days (time from onset to maximum disability)	5.6	3.9
	Frequency	Percentage
Gender Distribution		
Male	56	56%
Female	44	44%
Clinical Presentations of GBS		
Bulbar involvement	39	39%
Neck weakness	43	43%
Limb weakness	95	95%
Facial nerve involvement	49	49%
Type of neuropathy		
Axonal	56	56%
Demyelinating	44	44%
Frequency of Patients Requiring Mechanical Ventilation		
Required	13	13%
Not required	87	87%

Among those who required mechanical ventilation, at the time of presentation, all had limb weakness (100%), 11 out of 13 had facial weakness (84.6%), nine out of 13 had neck weakness (69.23%), eight out of 13 had bulbar involvement (61.5%), and three out of 13 had respiratory muscles involvement as measured by FiO2. All 13 patients attained maximum disability within five days. Out of those 13 ventilated patients, nine were male and four were female and five were below 40 years while eight were above 40 years of age. The clinical parameters of patients in the ventilated group are listed in Table 2.

**Table 2:** Clinical parameters of patients among ventilated group (n=13) Fraction of inspired oxygen: FiO2

	Frequency	Percentage
Gender		
Male	9	69.2%
Female	4	30.7%
Clinical features		
Limb weakness	13	100%
Facial weakness	11	84.6%
Neck weakness	9	69.23%
Bulbar weakness	8	61.5%
Respiratory muscle involvement	3	23.07%
Time from onset to peak disability < 5 days	13	100%
Age group		
< 40 years	5	38.4%
>40 years	8	61.5%
FiO2 at the onset		
FiO2=0.21	10	76.9%
FiO2>0.21	03	23.07%

When clinical presentations were compared in patients who required mechanical ventilation and those who did not, all clinical presentations showed a significant difference except limb weakness (Table 3).

**Table 3:** Stratification of clinical presentation in patients of GBS who need and who do not need mechanical ventilation GBS: Guillain Barre Syndrome

Clinical Presentations		Required Mechanical Ventilation	Did not require mechanical ventilation	P-value
Bulbar involvement	Yes	13	26	0.000
	No	0	61	
Neck weakness	Yes	13	30	0.000
	No	0	57	
Limb weakness	Yes	13	82	1
	No	0	05	
Facial nerve involvement	Yes	13	36	0.000
	No	0	51	
Time from onset to peak disability <5 days	Yes	13	26	0.000
	No	0	61	

## Discussion

Guillain Barre Syndrome (GBS) is one of the most common causes of acute flaccid quadriparesis. It is also one of the most common causes of neuromuscular respiratory failure, with patients requiring mechanical ventilation [1]. Respiratory failure contributes to significant morbidity and mortality in patients with GBS and is linked to a worsening of outcomes and long-term functional prognosis [13]. Respiratory compromise in GBS is linked to many factors. Upper airway compromise and weakness of pharyngeal and laryngeal muscles lead to difficulty in clearing secretions and airway maintenance, thereby also increasing the chances of aspiration. Weakness of the inspiratory and expiratory muscles of respiration leads to poor lung compliance, microatelectasis, hypoxemia, and increased risk of infections due to poor coughing ability. Pulmonary complications are also compounded by immobility, intensive unit stay, and mechanical ventilation.

Many clinical parameters have been extensively studied to predict respiratory failure in patients with GBS, as timely ventilation may change the course and prognosis for the patient. Rapid disease progression, bilateral facial palsy, autonomic dysfunction [14], and time from onset to admission less than seven days are some of these predictors. Respiratory function may be compromised much before signs of ventilatory insufficiency are obvious, and the serial assessment of ventilatory parameters is mandatory to determine the need for endotracheal intubation and mechanical ventilation [15]. Single breath count is a simple clinical bedside parameter to monitor lung function [16]. Regular assessment of patients with these simple bedside measurements should continue until clear and sustained improvement is observed.

The results of our study suggest that the need for mechanical ventilation should be anticipated in patients who have bulbar, facial, and neck weakness at the onset.

A consecutive series of 100 patients diagnosed with GBS admitted to the Neurology Department were studied. The mean age of our patient population was 48.1+/-8.01. 56% were males and 44% were females. A study conducted at Islamabad in 2014 showed the mean age of 33.83+/-16.9 years and a higher male preponderance, i.e, 68.48% [17]. The mean duration of symptoms from the time of onset to peak disability was 5.6+/-3.9 days. A duration of less than seven days is associated with a poor outcome [14]. The most common clinical presentation of GBS was limb weakness (95%) followed by facial nerve involvement (49%), neck weakness (43%), and bulbar involvement (39%), which is almost comparable to other studies [18].

In our study, 56% of cases had the axonal type of neuropathy while 44% had the demyelinating variety upon electrophysiological testing. The frequency of the axonal variant is higher in our patient population as compared to two other studies previously done in Pakistan, which showed a frequency of 40% and 31%, respectively [3,11].

Out of 100, 13% of patients ultimately required mechanical ventilation, which is less in comparison with other studies. Durand MC et al studied 154 patients and 22% required ventilator support [19], while 39% of patients required mechanical ventilation in a study conducted by Paul et al. [20].

Among the ventilated group (n=13) at the time of admission, all (100%) had limb weakness, 84% had facial weakness, 69% had neck weakness, and 61% had bulbar involvement. Only three (23%) out of 13 patients who later required ventilatory assistance had initial respiratory muscle involvement assessed by counting in a single breath and increased FiO2 (>0.21). Ventilator requirement is higher among males and those above 40 years of age. Old age is another predictor of poor outcome [21]. All 13 patients attained maximum disability in less than five days from onset of symptoms.

In our study, the frequency of different clinical presentations was 95% for limb weakness, 49% for facial weakness, 43% for neck weakness, and 39% for bulbar involvement. When compared to other studies, limb weakness was the initial symptom in 34 (94.4%) of 36 patients, an almost similar percentage [22]. In a study by Winer et al. [23], 53% had bilateral facial palsy, which is slightly higher than our results. Bulbar palsy was the most common (49.2%) in one study [24], which differs from our study.

The chi-square test was applied to compare the clinical presentations in patients who required mechanical ventilation and who did not. A p-value less than equal to 0.05 was taken as significant. Our study identified a shorter duration of symptoms from onset to lowest Medical Research Council (MRC) score (p-value < 0.000), facial weakness (p-value<0.000), neck weakness (p-value<0.000), and bulbar involvement (p-value<0.000) as significant predictors of mechanical ventilation in patients with GBS. A study conducted by Nabi et al. identified bulbar weakness and autonomic dysfunction as independent predictors of mechanical ventilation [17]. Lawn ND et al. [25] showed that progression to mechanical ventilation was highly likely to occur in those patients with rapid disease progression, bulbar dysfunction, bilateral facial weakness, or dysautonomia. Only three out of 13 patients (23%) among ventilated group showed signs of respiratory muscles involvement at admission; rather they had facial, neck, and bulbar weakness in higher percentages at the onset. This finding shows that there may not be respiratory comprise at the onset, but by the presence of other clinical presentations like facial, neck and bulbar involvement, we can predict the ventilatory requirement.

The classical signs of respiratory distress occur too late to serve as guidelines for management, and measurements of vital capacity and static respiratory pressures are useful to determine the best times for starting and stopping mechanical ventilation. However, this facility is not available in most neurology wards of the developing world.

Our results may allow the identification of GBS patients at admission who are likely at high risk of the requirement of ventilation in the course of the illness. These factors alone or in combination may not only necessitate immediate respiratory support but may strongly warn of an impending ventilation requirement and, therefore, aid in timely referral to tertiary care centers, thereby improving the care and outcomes. We believe that it is essential to identify the clinical presentations that can predict the need for mechanical ventilation support at admission in order to enable timely and appropriate care for patients with GBS. Here lies the significance of clinical examination, especially in those setups where advance facilities are not available.

The fact that this study was conducted at a single tertiary medical center limited the strength and generalisability of the study’s results. Future systematic studies with a larger sample size should be carried out to elucidate the overall nature of the clinical presentation experienced by patients with GBS, as well as its associated complications.

## Conclusions

In this study, the commonest presentation of GBS was limb weakness. Thirteen percent of patients required mechanical ventilation. The course of patients with GBS leading to mechanical ventilation can be predicted on the basis of clinical parameters. This study identified that a shorter duration of symptoms from onset to peak disability, bulbar involvement, neck weakness, and facial nerve involvement are significant independent predictors of mechanical ventilation. In our clinical setup, this information can guide us regarding the need for mechanical ventilation, which would be helpful in the early management of these patients by admitting them in the intensive care unit and preparing for elective intubation. These timely simple measures can decrease morbidity and mortality statistics.
